# Exploring Wellbeing and Creativity Through Collaborative Composition as Part of Hull 2017 City of Culture

**DOI:** 10.3389/fpsyg.2019.00548

**Published:** 2019-03-22

**Authors:** Caroline Waddington-Jones, Andrew King, Pamela Burnard

**Affiliations:** ^1^ School of Music, University of Hull, Kingston upon Hull, United Kingdom; ^2^ Faculty of Education, University of Cambridge, Cambridge, United Kingdom

**Keywords:** wellbeing, collaborative composition, older adults, creativity, Hull 2017, PERMA

## Abstract

Several studies have highlighted the positive effects of group music-making and have suggested that it may be the creative and social aspects of such activities, which have a positive effect on participants’ wellbeing. Collaborative composition offers strong examples of both aspects as participants work together to create new material. However, although it seems likely that participants’ influence over and ownership of the creative material contributes to these positive effects, studies have yet to examine these elements in detail. Through analysis of video observations, pre- and post-project interviews, video recall interviews, and questionnaires, this article aims to: (1) evaluate the impact of participation in collaborative composition workshops on the subjective and psychological wellbeing of older adults and (2) identify skills and approaches employed by the composer-facilitators in order to understand more fully the approach and skills employed to engage participants effectively in the creative process. This second aim is of particular interest given the current movement toward social prescribing and arts and health interventions in the UK. Analysis revealed that all dimensions of the PERMA framework for subjective and psychological wellbeing were present in this collaborative composition project. The specific nature of collaborative composition is considered in comparison with other forms of group musical engagement. For older adults, collaborative composition has much to offer as an activity encouraging social interaction with others with shared interests, increasing positive affect, and enhancing self-esteem. Analysis of workshop videos and interviews with composers identified various facilitation skills employed by the composers to establish safe creative space and to encourage participants to engage in the process of collaborative composition.

## Introduction

Since ancient times, humans have been fascinated with how to live a good life, with prominent thinkers such as Aristotle arguing that wellbeing is the overarching purpose of all human actions (Aristotle, 2004). In recent years, there has been increased research interest in wellbeing, as people seek to learn how they might lead healthier and happier lives. There has been much debate within the realm of positive psychology over how to define wellbeing, with researchers broadly conceptualizing two dimensions of wellbeing: subjective and psychological (e.g., Diener et al., 1999; Deci and Ryan, 2008; Proctor et al., 2009).

Our modern conceptualization of subjective wellbeing is rooted in the utilitarian hedonist philosophies of John Stuart Mill and Jeremy Bentham who both argued that pleasure is central to wellbeing. This dimension of wellbeing primarily concerns our subjective judgments of how satisfied we are with life (Diener, 1984) and emphasizes high positive affect and low negative affect (Bradburn, 1969; Kahneman et al., 1999). Subjective wellbeing involves the pursuit of happiness and is based on the principle that the more positive emotion we experience, the happier we will be (Seligman, 2002). By contrast, psychological wellbeing has emerged from Aristotle’s concept of *eudaimonia* and accentuates positive psychological functioning and human development (e.g., Rogers, 1961; Waterman, 1993). Various dimensions of positive psychological functioning have been proposed, including how we relate to other people, how independent we are, how we engage in various experiences, and how we find meaning in our lives (e.g., Seligman et al., 2005; Ryan et al., 2008).

More recently, positive psychologists have suggested that in order for humans to flourish fully, a balance of the elements of both subjective and psychological dimensions of wellbeing is required (Peterson et al., 2005; Sirgy and Wu, 2009; Seligman, 2010). Seligman (2010) has proposed a framework that incorporates elements of both dimensions of wellbeing: PERMA (Positive emotions, Engagement, Relationships, Meaning, Accomplishment). Positive emotions are the subjective wellbeing element of the PERMA model. The more positive affect or pleasure we experience, the happier we will be. Engagement in this context is closely connected to flow theory (Csikszentmihalyi, 1975) and refers to the experience of total immersion in an activity. The relationships’ dimension of the framework recognizes that humans are inherently social and that we thrive through our connections to and interactions with others. Having a clear sense of meaning or purpose in life seems to be a major component of human wellbeing and is the key to understanding motivation (De Muijnck, 2013). Finally, Seligman (2010) considers accomplishment to be a key motivation for many people. It is closely connected to goals and ambition, and it seems likely that gaining mastery or competence in a particular skill may increase self-esteem.

### Older Adults and Wellbeing

As people are now living longer lives, more research is being dedicated to understanding how we can flourish later in life. A review of the existing literature on older adults and wellbeing suggests that the Engagement and Relationships dimensions of PERMA may be particularly relevant. Cumming and Henry (1961) have proposed that as adults age, they disengage from society physically and socially, and that society also withdraws from older adults. Empirical research has suggested that older adults who are most connected report greater satisfaction with themselves and their lives—or greater subjective wellbeing (Bjorklund, 2011). Research has found that social engagement, in the form of social activities, productive activities, helping activities, formal and informal learning, and leisure, is positively correlated with older adults’ physical and mental health (Herzog et al., 2002), and that joint participation in leisure activities with members of one’s social network has been positively associated with life satisfaction (Holman and Jacquart, 1988).

Elsewhere, studies have found that participation in adult education has positive impacts on older adults’ psychological wellbeing (Schuller et al., 2002; Feinstein and Hammond, 2004; Hammond and Feinstein, 2006). This tended to take the form of increased self-esteem and seems likely to be related to the Accomplishment as well as the Engagement and Relationships dimensions of PERMA. Through mastering a new skill or acquiring knowledge, as well as engaging in a new activity and socializing with others, participants’ psychological wellbeing increases.

### Musical Engagement and Wellbeing

The field of Arts and Health in the UK is flourishing at present, particularly in light of the current emphasis on health promotion (see e.g., Coulter and Gordon-Nesbitt, 2016) and the formation of the *All-Party Parliamentary Group on Arts for Health and Wellbeing* in 2014. Most recently, in the UK, there has been a movement toward social prescribing, which advocates for engagement in arts-based activities to enhance wellbeing. Several studies have highlighted the positive relationship between musical engagement and both subjective and psychological dimensions of wellbeing (e.g., Hays and Minichiello, 2005; Ritchie and Williamon, 2011; Van Goethem and Sloboda, 2011; Croom, 2015). It has been argued that active rather than passive musical engagement tends to provide benefits for an individual’s psychological as well as subjective wellbeing (e.g., Csikszentmihalyi, 2002; Hallam et al., 2012; Creech et al., 2013). It seems likely that this may be due to the higher levels of engagement, the opportunities for self-expression, and the subsequent sense of accomplishment and clarity of identity inherent in active music-making.

Croom (2015) has proposed that the PERMA framework (Seligman, 2010) provides a useful lens through which to examine musical engagement and wellbeing. He cites evidence for musical engagement increasing positive affect (e.g., Laukka, 2007; Van Goethem and Sloboda, 2011; DeMarco et al., 2012), offering opportunities for deep engagement (e.g., Pates et al., 2003; Dietrich, 2004; Rogatko, 2009; de Manzano et al., 2010; Peifer, 2012), connecting with other people (e.g., Packer and Ballantyne, 2011; Procter, 2011; Schiepe-Tiska and Engeser, 2012; Koelsch, 2013; Rabinowitch et al., 2013; Ballantyne et al., 2014), bringing a sense of meaning or purpose to people’s lives (Frith, 1996; DeNora, 2000; Hays and Minichiello, 2005), and accomplishment (e.g., Hylton, 1981; Edgerton, 1994; Lee and Nantais, 1996; Hiscock et al., 2013; Klaphajone et al., 2013). Musical engagement, therefore, has the potential to contribute positively to all dimensions of wellbeing.

### Older Adults, Musical Engagement, and Wellbeing

Musicking in the lives of older people has begun to receive a lot of attention in recent years, with studies examining, for example, the benefits of everyday musical engagement for older adults (e.g., Hays and Minichiello, 2005), adults with dementia (e.g., Sixsmith and Gibson, 2007), and the impact learning a musical instrument can have on wellbeing for older adults (Perkins and Williamon, 2014).

#### Group Musicking

Wellbeing in relation to engagement in group music activities has been a particular focus of studies, as researchers seek to understand more fully this long-acknowledged positive impact of musicking with others (Small, 1999). Creech et al. (2013) investigated the relationship between engagement in group music-making activities and wellbeing according to the Basic Psychological Needs Scale (Deci and Ryan, 2008) and the CASP-12 measure of quality of life in later life (Higgs et al., 2003), finding that, even compared to other group activities, group music-making had a significant positive impact on participants’ self-reported wellbeing for the “purpose,” “control and autonomy,” and “social affirmation” components of these scales. Elsewhere, Hallam and Creech (2016) combined questionnaires with interviews and observations to examine the effects of participation in community music activities can have on older adults’ wellbeing. Again, compared to a control, the older adults participating in group music-making scored higher on quality of life instruments. Analysis of the qualitative data revealed benefits for health, emotional, and cognitive dimensions of wellbeing. Similarly, in a study characterizing collaborative and communal creativities in instrumental group learning and performance, Burnard and Dragovic (2014a,b) found the physicality of performance and levels of performance development to be sites for enhancing pupil wellbeing.

#### Collaborative Composition

Although such studies have considered that it may be the creative and social aspects of group music-making which particularly influence wellbeing, so far only one study (Habron et al., 2013) has explored the potential benefits of group music-making involving the collaborative creation of new musical material. The researchers found that the sense of control over musical materials, opportunities for creativity and identity formation, as well as social interaction with other participants and musicians were all beneficial for participants’ wellbeing. This is supported by other studies on collaborative composition, which have highlighted its potential for inducing flow (Macdonald et al., 2006) and have considered the collaborative aspect of the activity both socially and musically (Miell and MacDonald, 2000; Burland and Davidson, 2001; Hopkins, 2015; Williams, 2018).

Collaborative composition has not been considered yet through the lens of PERMA. However, as a form of active group musical engagement, and based on the evidence from these existing studies of collaborative composition and group music-making, it seems reasonable to suggest that collaborative composition may influence all dimensions of PERMA. While the participants in Habron et al.’s (2013) investigation of the relationship between collaborative composition and wellbeing had creative control over the music realized by the professional musicians involved in the project, they did not create the music themselves. Furthermore, no study has yet examined the facilitative skills required by composers in order to engage participants in collaborative composition. The present research therefore sought to explore the perceived impact of collaborative composition and group performance on diverse groups of people and to identify skills and approaches employed by the composer-facilitators.

### Hull 2017: New Music Biennial

As part of the program for Hull City of Culture 2017, five composer residencies took place over 4 months within various communities in the city as part of the PRS Foundation’s *New Music Biennial*. Designed to spark interest in collaborative composition and new music, the composers ran workshops with different groups of participants, including refugees and asylum seekers, travelers, older adults, primary school children, formerly homeless people, and vulnerable young people. The residencies culminated in two public performances at Hull City Hall, involving performances from each composer’s groups and a “supergroup” performance delivered by all the groups together.

Here, we present an evaluation of two of the composer residencies that sought to engage older people and primary school children: the first, a song-writing project bringing together older people living in retirement communities and children from a primary school in East Hull; the second, the creation of an ambisonic sound installation facilitated by a beatboxer and sound artist of international acclaim with members of choral group based in Hull. Two research questions will be addressed:

1. What was the perceived impact of engaging in collaborative composition in terms of psychological and subjective wellbeing?
2. What skills were required by the composers as workshop facilitators, engaging diverse groups of participants in collaborative composition?

## Materials and Methods

### Participants

The participants were two male, non-Hull-based, professional composers (Composer A and Composer B). Neither composer had a teaching qualification, but both had extensive experience of facilitating composition workshops, and this was the main reason that these two residencies were chosen out of the five for data collection and evaluation. Composer A worked with 25 members of a local choral group, a community choir whose membership changes on a project-by-project basis. Some members of the chorus were new for this project. Composer B worked with nine residents of two Hull-based homes from an independent Housing Association for older people in Hull. These residents formed a new group for the project. Later in the residency, they were joined by 25 years four pupils from a primary school in East Hull to collaborate for a final performance.

### Procedure

The two composers were interviewed at the start and at the end of their four-month residencies. Interviews were semi-structured and questions focused on the composers’ intentions for their residencies, their perception of possible challenges, and what they hoped the impact of the residency might be on the participants. Post-residency interviews followed a similar question structure but sought to reveal what had actually happened during the residency, what the challenges had been, and what the composers perceived to have been the impact of their residencies.

Two-hour workshop sessions with each composer were observed, and three of these observed sessions were also video-recorded. In addition to this observational data, recall interviews were conducted with two participants in Composer A’s residency using excerpts of the video data from one of the workshops. Finally, post-project questionnaires were administered to participants at one of the performance days at City Hall at the end of the project. These asked participants about their perception of the impact of the residency on them.

### Analysis

Interview, observational, and questionnaire data were reviewed, transcribed, and collated using *NVivo*. Thematic analysis was undertaken using an inductive approach modeled on grounded theory in which the aim of the analysis was to describe the data and theorize the findings. Themes were developed by collapsing, combining, or extending initial codes.

## Results and Discussion

In terms of the perceived impact of participating in the collaborative composition workshops and group performances on wellbeing, an inductive thematic analysis of the video-recall interview and post-residency questionnaire data revealed various themes. These themes were then considered in relation to the dimensions of PERMA, and some close connections were identified. Table 1 shows the theme and sub-themes, which emerged from the analysis.

**Table 1:** Themes and sub-themes for perceived impact of residencies according to participants.

Theme	Sub-theme
Enjoyment	
Togetherness	Social cohesion Musical cohesion
Empowerment	Engagement Meaning Accomplishment

### Enjoyment

Participants described or displayed “Enjoyment” of sessions. This theme seems to relate closely to the positive emotion dimension of PERMA. Examples of this theme from the post-residency questionnaires included comments such as “The project has been uplifting—I look forward to rehearsals” and “Uplifting—lifts the soul. Wonderful.” Reflecting on the residency, one of the video-recall interview participants explained:

It was fun, and I wanted to do more of it. To be part of different things … I have really enjoyed it and it definitely gave me a lot of energy and inspired me for the time in between the workshops and then afterwards. (Video-recall interview participant 1)

The positive emotion dimension of PERMA is connected to subjective wellbeing: the more positive affect we experience, the happier we will be. Since the participants found the workshops uplifting and fun, it seems likely that their participation in the workshops will have increased their subjective wellbeing.

### Togetherness

The theme “Togetherness” variously described bonding between participants (“social cohesion”) and coming together as a group musically (“musical cohesion”) and seems to be closely related to the Relationships dimension of PERMA.

#### Social Cohesion

In reflecting on their experiences of the residency, many participants emphasized their pleasure at making new friends and being together on a regular basis as a group. Comments from the Big Elastic Band on the post-residency questionnaires included: “I have made some great friends”; “I have really enjoyed getting out and being together with others in the group”; “I have taken away a spirit of willingness: wanting to learn and participate with everybody—children and adults.” Hull Freedom Chorus participants commented that they: “enjoyed being together”; “enjoyed meeting new people”; and “felt involved and happy with a changing group of singers.”

Despite being a group formed especially for this residency, it was evident that the Big Elastic Band members grew to value the social dimension that the collaborative composition workshops provided. This seems to connect closely to the Relationships dimension of the PERMA framework. The collaborative composition workshops offered participants a means of bringing together people with a shared interest in creating music who would not otherwise meet. The Big Elastic Band had been formed especially for the collaborative composition residency, and the 25 members of Hull Freedom Chorus who came together for this residency included several individuals who had not been a part of the group previously.

#### Musical Cohesion

In the post-project questionnaires, participants commented on the impact of the residency in terms of being able to “working with and listening to others” and “feeling able to make a creative contribution to the group.” While reviewing the video footage of a workshop, both video recall participants described their experiences of creating new sounds as a group:

I did have my eyes closed and I could hear everyone else. It amplified everything. Well that’s what the sea does, there’s all sorts of different sounds in the sea. It’s not just one sound, it’s all sorts of different sounds, and that’s what it sounded like to me. It just sounded like the sea. If you think of ripples of the sea going over pebbles. Each pebble is a different shape and making a different sound as it rushes back. That’s what it felt like to me. Everybody was doing it, like the sea ebbing away. It was a big sound and that’s what the sea is, it’s a big sound. (Video-recall interview participant 2)

Here, the participant describes a moment of group vocal improvisation in which the group are exploring the sounds of the sea. Research in group music-making has suggested that creating music together involves communication, cooperation, empathy, and ultimately may facilitate increased social cohesion (King, 2006; Seddon and Biasutti, 2009; Koelsch, 2013; Waddington, 2017). Since many participants in the present study reported that their participation in the residencies led to the formation of new friendships and a sense of group togetherness, the findings of this analysis seem to support the existing evidence in this area. Since collaborative composition involves co-creating new musical material rather than reproducing existing musical material, the various social and musical processes underlying this co-creation may be more pronounced.

In music educational contexts, Rusinek (2012) has suggested that when engaging in collaborative composition, participants operate in the “zone of proximal development” (Vygotsky, 1978) in which they co-construct musical understanding and new musical material. It is possible that these shared experiences of musicking and co-creation of musical material also contribute positively toward strengthening social relationships and an emerging sense of group identity.

### Empowerment

The theme of “Empowerment” broke down into sub-themes of “Engagement,” “Meaning,” and “Accomplishment”: dimensions of PERMA.

#### Engagement

In the Engagement sub-theme, participants described moments of total absorption in the creative process. When reviewing some workshop footage, one of the video-recall participants explained: “I found the sounds provoke the images for me. That’s what I liked about it. I was able not just to immerse myself in the sound which developed but the images came as well. I really appreciated that. I liked that” (video-recall interview participant 2). Such moments of absorption in these creative activities are characteristic of flow experiences (Csikszentmihalyi, 1975). Research has suggested that the more flow we experience, the happier we will be (Csikszentmihalyi, 2002). The finding of the present study that engaging in collaborative composition and group musicking may lead to flow experiences adds further weight to the existing evidence that group musical engagement in general and collaborative composition in particular can positively contribute to psychological wellbeing (e.g., Macdonald et al., 2006; Waddington, 2017).

#### Meaning

In their post-residency questionnaires, participants spoke about how the workshops had contributed to the structure of their weeks. One participant commented that the Big Elastic Band workshops were an important moment in their week to “get out and be together with others in the group.” Many participants wrote that they were looking forward to continuing to sing and compose together as a group. It seems likely that they had gained a sense of group identity and purpose.

#### Accomplishment

The post-residency questionnaires recorded many examples of accomplishment. Participants described various musical achievements: “confidence in singing (especially in a smaller group, it has proved you can sing, even though you are in your 80s)”; “musically—I now know how to make a song from scratch”; “stepping out of my comfort zone”; and “opened my mind to a greater variety of options for experimenting with sound and my voice.” One of the video-recall participants also noted that she “had a different feeling afterwards, like a sense of achievement because I did the drill and I got to hear myself being the drill.” A few participants also noted that this sense of accomplishment had led to, e.g., “greater confidence on a personal level.”

### Collaborative Composition and Wellbeing

There are many similarities between collaborative composition and other forms of active group music-making, such as ensemble playing. Both activities involve musical interaction, potential for experiencing flow, and socioemotional bonding. However, unlike ensemble playing, collaborative composition also involves creating new musical material. Consequently, there is a sense of group and individual ownership over the creative process in collaborative composition that may not be so prominent in ensemble playing activities. This aspect of ownership in collaborative composition also differs somewhat from group improvization in which the musical material is recorded in some form to enable reproduction. Capturing group members’ creative contributions in this way offers tangible evidence of accomplishment. This may enhance participants’ sense of achievement and increase self-esteem.

Another facet of collaborative composition, which differs from other forms of group musical engagement, is the potential for facilitating interaction and strengthening relationships between group members. Collaborative composition in the residencies evaluated for the present paper involved participants drawing on their life experiences or their personal memories of Hull as inspiration for the creation of songs or vocal soundscapes. As such, there were many opportunities for sharing personal experiences in each of the groups as part of the workshops as part of group discussions at the start of the creative process or through sharing ideas on the shaping of the musical material once composition had begun. It is likely that the sharing of these personal experiences facilitated socioemotional bonding between the group members. Below are the lyrics to one of the songs composed by the Big Elastic Band.

**Whisper**

I whisper in the night to my lost love

To dance with romance

To dance with romance

I took a chance

Forever in my heart

The love of my life

I wasn’t ready for you to go

Oh, how I loved you so

In the silence, you answer me

You say you needed to be free

Forever in my heart

The love of my life

Memories you left so clear

Will always keep you near

Through the heartache and pain

I know I will dance again

I know we will dance again some day.

(Lyrics to a song written by the Big Elastic Band)

This was one example of several sets of lyrics that members shared with the rest of the group. The lyrics to this song were written by one of the group members about a personal life experience. She shared the lyrics with the rest of the group who then all worked together, facilitated by Composer B, to create a melody for the song. The lyrics to this song are deeply personal to this group member. That she felt able to share her words with the rest of the group may be taken as evidence of the trust and the relationship that developed between members over the relatively short span of the residency. This adds further weight to the findings of the analysis of the interview and questionnaire data above that revealed that participants felt that their participation in the residencies had resulted in the development and strengthening of relationships with other members of the group.

These two elements—the recorded evidence of group or individual contributions and the opportunities for sharing personal experiences or ideas—seem to be unique to collaborative composition and relate closely to the Accomplishment and Relationships dimensions of PERMA. As with other forms of group music-making and as demonstrated in the analysis of the interview and questionnaire data here, collaborative composition can also contribute positively to the other dimensions of PERMA and, hence, to subjective and psychological wellbeing. For older adults in particular who may be less socially engaged or have lower self-esteem than younger adults (Orth et al., 2010), collaborative composition activities may positively contribute to the Relationships and Accomplishment dimensions of wellbeing.

Since there is compelling evidence in the rich, qualitative data analyzed here that collaborative composition had numerous benefits for older adults’ subjective and psychological wellbeing, it is important that we also seek to understand how these participants can be engaged successfully in collaborative composition. Encouraging participants to share ideas and engage in the creative process requires a lot of skill from the composers as facilitators move beyond their musical expertise. They must be able to build a rapport with the group members and establish a safe, non-judgmental space within the group that promotes this kind of engagement.

### Composers as Facilitators

These two composers were identified by the City of Culture managers in their post-project interviews as particularly good examples of composers working collaboratively to create material with participants. In their pre-project interviews, the composers identified two key challenges when considering how to approach engaging participants in collaborative composition during their residencies.

Composer A reflected on the challenge of coming in as an outsider and building trust with the participants in his residency in order to facilitate a creative process:

I think the first part of the challenge is building up a trust with people in Hull. I’m very conscious about being an artist that goes to a place where I’m not from …So, the whole thing for me is about really working with the people from Hull, to produce something that will not only go out, but will also hopefully remain in Hull. So I see myself more as a catalyst to facilitate a process that will come from the people that are already there. (Composer A, pre-residency interview)

The challenges of encouraging participants to engage in the creative process were also highlighted by Composer B, as he spoke about trying to bring people together:

And I suppose the challenge is always allowing people to be connected and easy with [the process of collaborative composition]…. It’s a very fractured community, you have people with all sorts of different abilities and they’re coming in, they’re coming out, so everything has to grow very organically. I think the challenges are really just about finding different ways that people can kind of connect to it, you know. As soon as they connect to it, it starts being part of what they are and they have a sort of sense of belonging to it and then that helps them grow from there. It’s just finding different ways of doing that …. The whole residency is about allowing all of those people to write their own stuff essentially. (Composer B, pre-residency interview)

This composer recognized the additional challenge that at least one of the groups of participants that he was facilitating was formed just to participate in his residency. As a newly formed group, there would be a period of “forming” (Tuckman, 1965) as members settled into the pattern of working together as well as working with the composer-facilitator. This presents a further challenge from the facilitator’s perspective in terms of engaging participants in a group creative process such as collaborative composition.

Common to both of these accounts is a sense that facilitating group ownership rather than composer ownership over the creative process and material produced was a priority for both composers. Composer A described himself as “a catalyst to facilitate a process that will come from the people” and Composer B spoke about participants’ sense of “belonging” and connection to the process. It was evident from these pre-residency interviews that these composers both set out to facilitate true collaborative composition rather than taking a more composer-directed approach to their residencies.

Analysis of the video data identified the facilitation skills and tools employed by the composers (see Table 2). The creation of safe space is critical for participants to feel comfortable engaging in a creative process (Rogers, 1993). Both composers used a range of skills to facilitate this engagement. Three themes were identified in the video data: “Confidence and Clarity,” “Facilitator-Group Dynamic,” and “Comfort.” Each theme had various sub-themes.

**Table 2:** Coding framework for facilitator skills.

Theme	Sub-themes
Confidence and clarity	Demonstrating expertise Modeling Precise explanation
Facilitator-group dynamics	Intimacy vs. authority Group ownership Composer-directed Praise and feedback Humor
Comfort	Comfort level Environment and physical needs

#### Confidence and Clarity

Both composers were coded as “Demonstrating Expertise” at regular intervals. These were moments were the composer-facilitators demonstrated their musical skills and served to remind workshop participants of the composers’ expertise and, consequently, encouraged a higher level of confidence in both the composer and the creative process. Both composers modeled techniques frequently rather than explaining verbally. The theme of “Modeling” often overlapped with “Demonstrating Expertise,” suggesting that Modeling was a way of explaining exactly what the composer wanted the group to do while also reminding them of the composer’s expertise. Ensuring a good understanding of instructions also contributed to the establishment of safe space. When verbal instructions were used, both composers were very clear in outlining ideas (coded as “Precise Explanation”). This seemed to minimize confusion and uncertainty and so contributed to establishing safe space and facilitating creative expression. This supports Macdonald et al.’s (2006) finding that the clarity of instructions is crucial for engaging participants in collaborative composition.

#### Facilitator-Group Dynamic

There were examples in both composers’ workshops of the composers taking on a more authoritative role, to guide the workshop, for example, and a more equal role with the participants, encouraging group members to contribute or take ownership. These moments were coded as “Intimacy vs. Authority.” Within this sub-theme was two further sub-themes: “Group Ownership” and “Composer-Directed.”

Group Ownership described moments when the composers placed control over the creative material into their group’s hands. This was supported by the video-recall interviews in which one participant commented that the composer had “given us that freedom. He’s given us that opening to say ‘Yes, I want to know what your opinions are’ not closed up and said, ‘You’ll do it my way.’” This sub-theme was balanced at times with “Composer Direction,” where composers would direct the creative process toward the requisite end product: the performances at City Hall.

To encourage contributions from the group, create a positive atmosphere, and offer reassurance and clarity, both composers offered “Praise and Feedback” to participants at regular intervals throughout the workshops. This seems to be in line with flow theory (Csikszentmihalyi, 1975), which suggests that frequent feedback is a condition for flow. Both composers offered encouragement, acknowledged effort, and praised participants at regular intervals during workshops.

Another tool both composers employed to cultivate a more egalitarian relationship with the group members and to encourage engagement was “Humor.” In his pre-residency interview, Composer B explained:

The thing about writing music on any level is that it’s like being in this brilliant playground where you are surrounded by lots of stuff, you know. So it’s just a question of putting things on top of each other and connecting things together. And it’s a real sense of play you know. And that’s, I think, what we try to do, we enable this sense of just pure, ridiculous fun in sticking stuff together. And then that leads somewhere. (Composer B, pre-residency interview)

This concept of play for both composers was central to engaging participants. During one of his workshops, composer A explained this to his participants:

Underneath it all it’s all about play. It’s all about playing. It’s like what happens when you are a kid and just sheer, just getting muddy just for the hell of it. Covered in dirt, wow. It’s about playing, and what I am trying to say to you is just try to play with stuff and see what happens, you know. That’s the most important thing is that we play. (Composer A, workshop 19 June 2017)

This finding supports existing research suggesting that overcoming participants’ fear of failure is crucial to engaging them in the creative process and in flow (Macdonald et al., 2006). Taking a playful approach to composition seemed to be strategies employed by both composers in their workshops. Humor seemed to be a mechanism for encouraging play during both composers’ workshops.

#### Comfort

Both composers emphasized voluntary participation at individual group members’ comfort levels: “Comfort Level.” By emphasizing the voluntary nature of participation, the composers empower the participants, giving them control over their level of engagement in the creative process. The composers also considered group members’ comfort on a more practical level. Examples included reminders to rehydrate, asking whether participants would prefer to sit, checking energy levels and the temperature of the room. This was coded as “Environment and Physical Needs.” This seems to relate to Maslow’s (1987) Hierarchy of Needs, since if participants’ basic needs in terms of physical comfort are not met, they will not be able to engage.

### The Role of the Composer-Facilitator

For these residencies, there was a balance between working toward a final product, a public performance, and emphasizing engaging in the creative process. Reflecting this challenge, there was a balance between composer-directed moments, and moments where the composers encouraged the group members to take ownership over the creative process. This way of working draws upon the composers’ expertise not only in composition but also in facilitating, and ultimately empowering, their participants.

## Conclusion

This study has added further evidence to the existing literature on the relationship between collaborative composition and wellbeing. Analysis revealed that participants in the 4-month collaborative composition residencies for Hull 2017 perceived a positive impact on their subjective and psychological wellbeing, with participants’ experiences being closely associated with all five dimensions of the PERMA framework (Seligman, 2010). It is interesting to note that the PERMA framework here emerged from an inductive analysis of the observational, video-recall interview, and questionnaire data rather than from a direct application of the framework to the data. The PERMA framework is beginning to be applied to highlight the benefits of musical engagement in various music educational and performance contexts (e.g., Lee et al., 2017; Ascenso et al., 2018). However, our work here suggests that there is much potential for this framework to be used as an evaluative tool for community music and music therapy projects as well.

The specific nature of collaborative composition has been considered in comparison with other forms of group musical engagement, and the potential for participants to experience a sense of achievement in identifying their own contribution to the group’s work as well as the potential for group discussion, particularly focusing on personal experiences, and the impact that may have on strengthening relationships between group members have been highlighted. For older adults, collaborative composition has much to offer as an activity encouraging social interaction with others with shared interests, increasing positive affect, and enhancing self-esteem.

While there is strong evidence here for the value of collaborative composition as an activity that may enhance wellbeing, the intersubjective process of collaborative composition is complex and requires a range of skills from the composer-facilitator in order to engage participants effectively. With the current interest in arts and health interventions and social prescribing, it is vital that consideration be given to the quality of provision of projects such as this. This article has begun to consider some of the skills required to facilitate effective collaborative composition workshops with older adults. The City of Culture managers and the authors had identified the two composer residencies reported here as being the most successful in engaging participants in collaborative composition. Analysis of interview and video data identified the various techniques employed by the composer-facilitators to establish a safe space for participants to feel able to engage in the creative process.

Future work should engage in practices of reconfiguring and re-reading what counts as generative forces of wellbeing and the physicality of collaborative composition and performance practice/engagement and to evaluate the legacy of collaborative composition and performance projects such as this in terms of participants’ continued engagement with music-making and the perceived impact on their wellbeing over time. The intention to keep engaging in music activities beyond this project was declared by most participants in post-project questionnaires and recall interviews. Many members of the Hull Freedom Chorus were keen to be involved in future projects exploring beatboxing, rap, or other less conventional styles. Funding has been awarded based on the success of this residency to support further work with the Big Elastic Band and continue their song-writing workshops. We intend to work with some of these participants over the coming months to evaluate their continued engagement in music-making beyond this project.

Nearly 20 years ago, in one of the earliest studies of children’s group music compositions, Morgan et al. (2000) argued that the principal element of task activity within groups is the dialog among members. It is surely high time we recognized the importance of continued engagement with such music-making in terms of impact on wellbeing because it allows for ways to think differently and produce, juxtapose, contrast, combine, and communicate ideas through collaborative composition.

## Data Availability

The datasets for this study will not be made publicly available because participants have not consented for their video and interview data to be made available.

## Ethics Statement

This study was carried out in accordance with the recommendations of “The University Code of Good Research Practice,” University of Hull Ethics Committee, with written informed consent from all subjects. All subjects gave written informed consent in accordance with the Declaration of Helsinki. The protocol was approved by the University of Hull Faculty of Arts, Culture, and Education Ethics Committee.

## Author Contributions

CW-J collected the data, analyzed, and drafted the manuscript. AK is a principal investigator and designed the study. AK and PB revised the manuscript. PB consulted at each stage of project.

### Conflict of Interest Statement

The authors declare that the research was conducted in the absence of any commercial or financial relationships that could be construed as a potential conflict of interest.
